# Human brain slice cultures: translational applications and ethical considerations

**DOI:** 10.26508/lsa.202403160

**Published:** 2025-10-09

**Authors:** Kevin Joseph, Ioannis Vasilikos, Juergen Grauvogel, Mukesch Johannes Shah, Peter C Reinacher, Julia M Nakagawa, Ute Häussler, Jakob Straehle, Nicolas N Neidert, Panagiotis Fistouris, Matthias Schneider, Steven A Sloan, Tobias Weiss, Volker A Coenen, Oliver Schnell, Andreas Vlachos, Marco Prinz, Ulrich G Hofmann, Jürgen Beck, Philipp Kellmeyer, Vidhya M Ravi

**Affiliations:** 1 Faculty of Medicine, University of Freiburg , Freiburg, Germany; 2 Department of Neurosurgery, Medical Center–University of Freiburg , Freiburg, Germany; 3 Laboratory of Neuro-Engineering, Medical Centre University of Freiburg , Freiburg, Germany; 4 3D-Brain Models Lab for Neurodegenerative Diseases, Medical Centre University of Freiburg , Freiburg, Germany; 5 Institute of Neuropathology, Medical Center—University of Freiburg , Freiburg, Germany; 6 Signalling Research Centre BIOSS and CIBSS, University of Freiburg , Freiburg, Germany; 7 Neuroelectronic Systems, Medical Center—University of Freiburg , Freiburg, Germany; 8 German Cancer Consortium (DKTK), Partner Site Freiburg, Freiburg, Germany; 9 Department of Neuroanatomy, Institute of Anatomy and Cell Biology, Faculty of Medicine, University of Freiburg , Freiburg, Germany; 10 Center BrainLinks-BrainTools, University of Freiburg , Freiburg, Germany; 11 Center for Basics in Neuromodulation (NeuroModulBasics), Faculty of Medicine, University of Freiburg , Freiburg, Germany; 12 Center for Advanced Surgical Tissue Analysis (CAST), University of Freiburg , Freiburg, Germany; 13 Translational Epilepsy Research, Department of Neurosurgery, Medical-Center, University of Freiburg , Freiburg, Germany; 14 Berta-Ottenstein Programme, Faculty of Medicine, University of Freiburg , Freiburg, Germany; 15 Data and Web Science Group, School of Business Informatics and Mathematics, University of Mannheim , Mannheim, Germany; 16 Fraunhofer Institute for Laser Technology, Aachen, Germany; 17 Freiburg Institute of Advanced Studies (FRIAS), Freiburg, Germany; 18 Department of Human Genetics, Emory University School of Medicine, Atlanta, GA, USA; 19 Emory Center for Neurodegenerative Disease, Emory University School of Medicine, Atlanta, GA, USA; 20 Comprehensive Cancer Center Erlangen-EMN (CCC ER-EMN), Erlangen, Germany; 21 Department of Neurosurgery, Uniklinikum Erlangen, Friedrich-Alexander-Universität Erlangen-Nürnberg, Erlangen, Germany; 22 Department of Neurosurgery, University Hospital Bonn , Bonn, Germany; 23 Brain Tumor Translational Research Group, University Hospital Bonn , Bonn, Germany; 24 Department of Neurology, Clinical Neuroscience Center, University Hospital Zurich and University of Zurich, Zurich, Switzerland; 25 Department of Neurosurgery, Clemens Hospital Münster , Academic Hospital of Münster University, Münster, Germany; 26 Department of Stereotactic and Functional Neurosurgery, Medical Center of Freiburg University , Freiburg, Germany; 27 Centre for Deep Brain Stimulation, Medical Centre of Freiburg University, Freiburg, Germany

## Abstract

Human organotypic brain slice cultures have emerged as a pivotal tool to study the complexities of the human brain. Human organotypic brain slice cultures preserve the structural integrity, cellular diversity, and vascular networks of living brain tissue, maintaining in vivo characteristics. This advancement enables accurate temporal modeling of neurological diseases and facilitates precise experimental manipulations, accelerating therapeutic development. However, their use raises important ethical and philosophical considerations, including issues of donor consent and the potential for neural activity that prompts questions about consciousness. This study outlines these emerging concerns, emphasizing the need for guidelines that balance scientific innovation with ethical responsibility, particularly in relation to donor consent, transparency, and long-term use of living human tissue.

## Abstract (precis)

This review outlines the scientific utility and ethical imperatives of human brain slice cultures, calling for global ethical standards in ex vivo neuroscience research.

## Introduction

The adult human brain is among the most complex functional systems in biology, comprising ∼80 billion neurons and 40–50 billion glial cells. This immense cellular population contributes to its status as possibly the most intricate functional structure discovered in the universe (1). Across mammalian species, brain size can vary by up to 100,000-fold, highlighting the remarkable diversity within this class. Given this complexity, creating accurate and physiologically relevant models to study human brain function and pathology has become essential (2). Traditional models, like rodent systems and dissociated neuronal cultures, have provided invaluable insights but often fall short in replicating the architecture and network dynamics of the human brain. Findings from animal models do not always predict human outcomes, so models that more closely resemble human brain tissue are “needed to” better reflect human-specific pathophysiology and improve clinical relevance (2).

Early human brain model experiments used acute human brain slices, which are thin sections of freshly resected brain tissue that can be maintained in vitro for several hours after surgical removal. These acute slices allow direct investigation of human neuronal physiology shortly after removal and have yielded insights into human synaptic function and pathology (3, 4). For example, researchers have performed electrophysiological recordings and observed synaptic plasticity in acute slices from epilepsy surgery patients (5). However, the experimental window with acute slices is brief, which limits prolonged or repeated interventions (5, 6). Although important data can be obtained from acute preparations, long-term studies (such as extended drug exposures or disease progression modeling) are not feasible in such a short time frame. Over the past decades, significant efforts have been made to extend the viability of brain slices in vitro (7, 8). A breakthrough came in the 1960s with improved tissue culture techniques: the introduction of roller–drum (9) and interface culture systems enhanced oxygenation and nutrient supply, markedly extending slice lifespan. Optimizations in culture media and conditions further supported neuronal survival and preserved glial cells and vasculature (10, 11). These advancements enabled longer term experiments on human brain tissue ex vivo. It is now possible to obtain viable human brain tissue from surgical resections and even postmortem donations for research purposes, enabling the creation of human organotypic brain slice cultures (HBSCs) that can be maintained for days to weeks (7, 12, 13, 14, 15, 16 *Preprint*, 17, 18).

**Figure 1.**
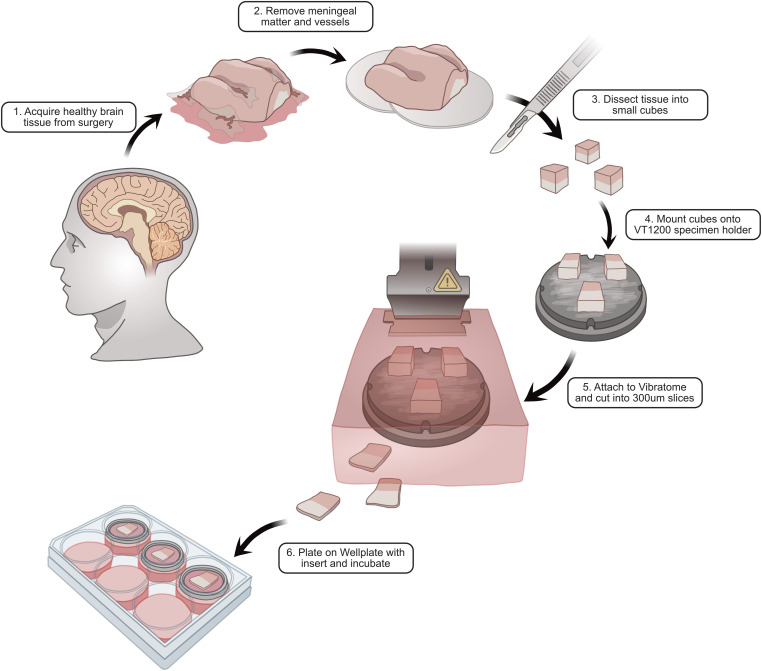
Workflow for processing freshly resected human cortical specimens during surgery (as already established at the Department of Neurosurgery, Medical Center–University of Freiburg). This workflow shows the steps from surgical resection of brain tissue to the preparation and culturing of acute and organotypic human brain slices, including tissue transport, slicing, and maintenance in culture media. Illustration created by Nathan Conrad.

In current practice, researchers verify the vitality of these long-term slices using both electrophysiological and biochemical assessments. For instance, sustained neuronal firing and synaptic responses are confirmed via patch-clamp or multielectrode array recordings, whereas viability assays (such as live/dead cell staining or lactate dehydrogenase release) measure the proportion of living cells (19). Slices that retain robust electrical activity and minimal cell death for multiple weeks in vitro are considered to have high culture fidelity (19). Fresh surgical tissue offers several advantages over postmortem tissue in this context (see Box 1), and dedicated protocols have been established to process freshly resected brain specimens into organotypic cultures (Fig 1).

Box 1 Comparison of tissue sources for human brain slice cultures.

Fresh tissue (surgical resection).

• *Pros*: Enhanced viability and functional activity, crucial for long-term studies and accurate physiological assessments. The native cellular architecture and microenvironment are better preserved, allowing more faithful studies of neural and glial interactions.
• *Cons*: Limited availability—access depends on surgical schedules and the specific pathologies requiring surgery. Tissue often comes from patients with neurological conditions (e.g., tumors or epilepsy), which may influence the generalizability of findings.

Postmortem tissue.

• *Pros*: Greater accessibility—postmortem donations can provide larger quantities of tissue, including from individuals without neurological disease. Allows studies across different ages and conditions that might not require surgery.
• *Cons*: Reduced viability—any delay between death and tissue preservation (postmortem interval [PMI]) can lead to degradation of RNA, proteins, and cellular structure, compromising tissue quality. Even short PMIs can induce cellular changes that affect experimental interpretations. In addition, lack of blood flow and oxygen during the PMI can alter physiology, so outcomes must be interpreted cautiously.

**Figure 2.**
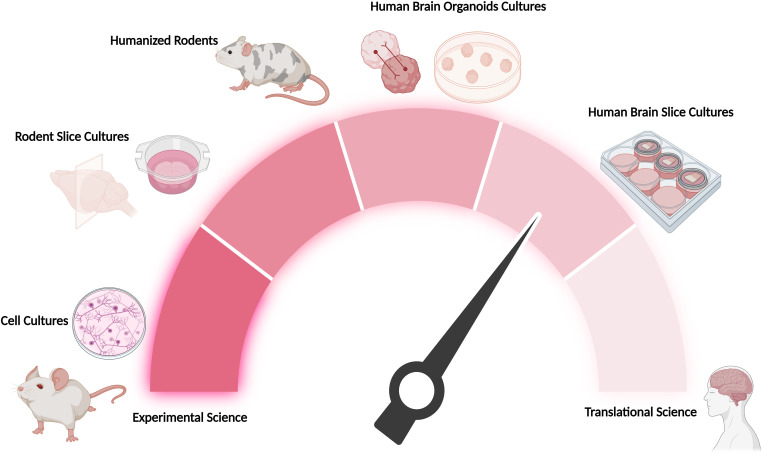
Simplified schematic illustrating various brain model systems and their fidelity to human in vivo brain tissue. This figure provides an overview of how closely different experimental models—ranging from cell cultures and organoids to human ex vivo slice cultures and in vivo animal models—recapitulate the cellular complexity and circuitry of the human brain. Created with BioRender.com.

Access to viable human brain tissue has opened new avenues for research. Box 2 summarizes common neurosurgical procedures that can provide fresh brain tissue for slice cultures. These surgical specimens offer valuable opportunities to study human brain biology under both relatively healthy conditions and specific disease states (e.g., epilepsy or tumor) (20, 21). These tissues, when cultured as organotypic slices, preserve the brain’s 3D cytoarchitecture and cell-type diversity. By maintaining the intricate architecture and cellular interactions of human brain tissue, organotypic slice cultures serve as an invaluable platform for studies that bridge the gap between traditional in vitro models and in vivo research (Fig 2).

Box 2 Access to fresh human brain tissue.

Common surgical indications that provide fresh human brain tissue samples include the following:

• Epilepsy surgery: Resection of epileptogenic brain regions to control seizures, often yielding adjacent cortical tissue that can be cultured.
• Tumor removal: Excision of primary brain tumors (such as gliomas) or metastatic lesions, where surrounding brain tissue removed for surgical margins can be used for slice cultures.
• Intracerebral hemorrhage evacuation: Removal of blood clots or hemorrhagic tissue to relieve intracranial pressure in stroke or trauma patients, occasionally providing cortical tissue fragments.
• Ventricular shunt placement: In hydrocephalus treatment, a small cortical biopsy is sometimes taken when inserting a ventricular shunt catheter, and this tissue can be repurposed for research.
• Stereotactic biopsy—MRI-guided needle sampling of deep or eloquent brain regions for diagnostic purposes; core specimens provide millimeter-scale tissue for live‐slice cultures and single-cell analyses.
• DBS lead placement and revision—Lead placement or electrode trajectory adjustment for lead replacement in movement disorder therapy; cortical access during lead positioning/repositioning yields small but viable samples for mechanistic and translational research.

In essence, human organotypic slices allow more physiologically relevant experimentation than dissociated cell cultures, while avoiding some complexities of in vivo studies. Researchers have developed biobanks of such cultures, enabling multiple investigations on the same human tissue, which can accelerate the development of effective treatments (13, 15, 21, 22).

Given their unique ability to preserve native cytoarchitecture, cell-type diversity, and human-specific physiology, HBSCs have emerged as a versatile platform with translational relevance across multiple domains of neuroscience. Beyond serving as static models, they now facilitate mechanistic discovery, therapeutic development, and patient-specific interventions. In the following sections, we highlight the principal research areas where HBSCs have been deployed effectively and discuss how their application continues to transform our understanding of human brain function, disease, and treatment.

## Research Applications of Human Ex Vivo Brain Slice Cultures

HBSCs are being applied in diverse research areas. This section highlights major use cases, from tumor biology and drug testing to modeling neurological disorders and infections, underscoring the broad scientific potential of these cultures.

### Tumor biology and neuro-oncology

HBSCs have become a pivotal platform for studying glioblastoma and other CNS tumors in a human-relevant microenvironment. Unlike cell lines or animal models, HBSCs preserve the three-dimensional architecture, cytoarchitecture, and cellular diversity of human brain tissue, enabling highly translational insights into tumor biology and therapeutic responses.

Invasion and tumor–microenvironment interactions: Human-to-human invasion models, introducing patient-derived glioblastoma (GBM) cells into human brain slices, allow direct observation of tumor infiltration along white-matter tracts and blood vessels, closely mimicking in vivo behavior (12, 15, 22, 23). This permits a detailed examination of the mechanisms underlying tumor infiltration and the identification of factors that facilitate invasive behavior in human tissue context. HBSCs also retain resident astrocytes, microglia, neurons, and extracellular matrix, enabling investigation of tumor–host interactions that drive progression and resistance. Notably, GBM cells have been shown to reprogram surrounding glia into immunosuppressive phenotypes (15, 18). One study demonstrated that treatment with the gap junction inhibitor meclofenamate (MFA) not only killed tumor cells but also disrupted their intercellular communication networks, highlighting how HBSCs can reveal therapeutic effects on the multicellular architecture of the tumor microenvironment (24).

Therapeutic and immunotherapeutic testing: HBSCs are increasingly used as ex vivo platforms to test drug efficacy, particularly for glioma. Temozolomide and other agents demonstrate predictable antitumor effects, and the presence of blood–brain barrier elements within slices enhances translational relevance. Beyond traditional chemotherapeutic agents, HBSCs have also become a testbed for next-generation immunotherapeutic strategies (15). Slices have been used to analyze how GBM cells secrete cytokines (like IL-10, TGF-β) that suppress T-cell activity and promote regulatory T cells, contributing to immune escape (21). These insights are guiding the development of therapies to counteract immunosuppression. We can apply emerging treatments, such as checkpoint inhibitor drugs or CAR T cells engineered to recognize GBM, onto tumor-infiltrated slices to assess their efficacy in inducing immune-mediated tumor cell killing.

### Neurological disease modeling

Beyond neuro-oncology, HBSCs have proven useful in modeling various neurological and neurodegenerative conditions. Slices prepared from epileptic brain tissue, for instance, can exhibit spontaneous epileptiform activity, providing a platform to study seizure mechanisms and test antiepileptic drugs ex vivo (25). Similarly, slices have been used to model neurodegenerative proteinopathies by introducing pathologic proteins or observing the progression of pathology in patient-derived tissue (26). Another emerging application is the use of postmortem human brain slice cultures from donors with neurodegenerative diseases. Recent work has shown that even postmortem adult brain tissue can exhibit forms of synaptic plasticity when cultured organotypically (27).

This suggests that slices from donors with conditions like Parkinson’s disease or dystonia could be used to investigate disease processes and screen neuroprotective compounds with the actual patient tissue, which retains relevant age-related changes and disease markers. Another avenue that opens is the surgical treatment of psychiatric disorders like major depression, obsessive–compulsive disorder, and Gilles de la Tourette syndrome, for which surgical therapies like deep brain stimulation (DBS) are emerging.

### Infectious disease and neuroimmunology

In parallel with chronic neurological diseases, HBSCs also provide a powerful platform to study acute perturbations to neural homeostasis, such as those triggered by infectious agents. For ethical and practical reasons, many human-specific pathogens cannot be studied in living patients or standard animal models. Organotypic slice cultures bridge this gap. For example, human fetal organotypic brain slice cultures (hfOBSCs) have been developed to model herpes simplex virus (HSV) infection in central nervous system tissue. Using brain slices from second-trimester human fetal tissue, researchers maintained a variety of brain-resident cells (neurons, astrocytes, microglia, oligodendrocytes) in culture for up to two weeks and infected them with HSV-1 or HSV-2 (28). The infected slices exhibited hallmarks of herpes encephalitis: the virus preferentially infected neurons and astrocytes and triggered programmed cell death (via necroptosis) in these cells, along with a robust inflammatory cytokine response (28). This slice-based infection model closely recapitulated human disease pathology and has proven valuable for testing antiviral drugs in a setting that mirrors human brain infection. Beyond HSV, similar ex vivo approaches have been applied to other neurotropic viruses (22). For instance, researchers have used fetal human brain slices to study emerging viruses: one recent study demonstrated that the Usutu virus, a mosquito-borne flavivirus, infects human slices and causes cellular changes that partially resemble those seen with Zika and West Nile virus infections. Such findings indicate that a broad range of viral pathogens can be investigated using organotypic human slices (29). This is particularly useful for viruses that have limited tropism in animal models or where human-specific immune responses are of interest. HBSCs thus provide a platform to examine viral replication, cell-type vulnerability, and immune reactions in genuine human brain tissue.

### Gene therapy/oncolytic viruses

In addition to modeling natural infections, HBSCs can be used to test viral therapies. For example, oncolytic viruses (genetically engineered viruses that selectively kill tumor cells) are being developed for glioblastoma treatment (30). Human brain slice cultures can serve as a preclinical testing ground for these agents: a candidate oncolytic virus can be applied to a GBM-infiltrated slice to observe whether it infects and destroys tumor cells effectively, and to monitor any off-target infection of healthy brain cells. Similarly, gene therapy vectors (like AAV-based vectors for neurological disorders) can be evaluated in HBSCs to ensure they transduce the intended cell types and do not cause undue toxicity in human neural tissue (31). Using HBSCs in this way accelerates the development of therapies by enabling human-specific efficacy and safety testing early in the research pipeline.

As the capabilities of HBSCs expand, allowing for sophisticated modeling of brain function, disease, and therapy in human tissue, a parallel discourse on the ethical, legal, and social implications becomes indispensable.

## Ethical Considerations in HBSC Research

This section outlines the foundational ethical requirements governing research with human brain slice cultures (HBSCs), including informed consent, institutional oversight, and donor privacy, before addressing the unique ethical challenges posed using living human brain tissue.

### Standard ethical requirements

Research using human brain slice cultures (HBSCs) must meet the established ethical standards for human biological material: informed consent, institutional ethics committee approval, and protection of donor privacy. Consent must be obtained from patients or next of kin, clearly outlining the research purpose, use of tissue, risks, benefits, and donor rights, including withdrawal (32). Broad or dynamic consent models, approaches that allow ongoing, flexible, and interactive consent processes (often via digital platforms), enabling participants to adjust their preferences over time, are increasingly used to support long-term and exploratory research while maintaining donor agency. Ethics committees (IRBs) review all protocols to ensure tissue acquisition, usage, and data handling meet ethical criteria (33). Confidentiality must be safeguarded using coded or de-identified samples, with data storage compliant with regulatory frameworks such as GDPR (EU) and HIPAA (US). Importantly, researchers must not influence clinical decisions: brain tissue should only be collected from surgeries already indicated for therapeutic or diagnostic purposes, with no alteration to clinical care for research objectives. The surgical consent, outlining the medical procedure and its clinical rationale, must be obtained solely by the attending neurosurgeon. Separately, a dedicated research consent should be obtained by the scientist or clinician responsible for the HBSC experiments, ensuring that the donor (or next of kin) understands how the tissue will be used for research purposes. This dual-consent structure ensures clear separation of clinical and research responsibilities. Institutional safeguards should reinforce this separation to uphold the primacy of patient care and foster public trust (34).

### Specific ethical challenges: consciousness and neural complexity

One unique ethical challenge raised by HBSCs is whether ex vivo brain tissue could exhibit organized neural activity consistent with rudimentary consciousness (32). Although this question has been widely debated, especially in the context of brain organoids, there is currently no scientific evidence that cultured slices display features associated with conscious experience (35, 36). Most slices lack structural and functional integration, including long-range connections and thalamocortical loops thought to be necessary for consciousness. The Integrated Information Theory (IIT) posits that consciousness arises from a system’s capacity to integrate information, quantified by a metric (Φ). Brain slices likely exhibit very low Φ values compared with intact brains. Another model, the Global Neuronal Workspace theory, proposes that consciousness requires widespread information broadcasting across brain networks, dependent on intact cortical–subcortical circuits that are absent in HBSCs. Many neuroscientists argue that brainstem arousal systems and thalamic loops are prerequisites for conscious experience. Based on these criteria, HBSCs are highly unlikely to be conscious. Although we mention Integrated Information Theory (IIT) as one framework, it remains controversial and is one of several perspectives on consciousness. The Global Neuronal Workspace theory similarly holds that consciousness requires widespread information broadcasting across intact long-range connections and cortical–subcortical loops, which are absent in HBSCs and organoids (37, 38). Along with the lack of brainstem arousal systems and thalamocortical circuits, these limitations make it highly unlikely that HBSCs possess consciousness. A recent ethical assessment supports this view, concluding that current cerebral organoids and ex vivo brain tissues do not yet reach the complexity required for consciousness, while noting that future advances may shift this boundary (36).

### Global ethical framework and consent governance

As the field of HBSCs progresses toward increasingly complex applications in neuroscience and translational medicine, there is a pressing need for a globally harmonized ethical framework. We propose that an international norm-setting body, such as UNESCO, the Council for International Organizations of Medical Sciences (CIOMS), or the WHO, convene expert stakeholders to establish dedicated guidelines governing the acquisition, use, long-term maintenance, and cross-border sharing of living human brain tissue. Central to this framework should be robust recommendations for informed consent that reflect the unique bioethical status of HBSCs: viable, metabolically active human brain samples that may be preserved over extended periods, reused across multiple experiments, and integrated with multi-omic and imaging datasets. Consent protocols should explicitly communicate to donors the scope of tissue viability, potential for long-term storage, experimental reuse, and international data linkage. In addition, such a framework should include provisions for equitable access, benefit sharing, and governance mechanisms to ensure ethical oversight across jurisdictions.

### Moral status and stewardship of living tissue

Another ethical aspect unique to HBSCs is the question of moral status and long-term stewardship of living human brain tissue. Slices maintained for weeks in vitro are not merely inert samples; they are functioning pieces of human brain. This situation challenges our traditional understanding of the boundary between life and death—for example, in the case of postmortem tissue, one might ask if a part of a person’s brain is still “alive” in a meaningful way in the petri dish, does it carry any residual moral status? Some ethicists have noted that keeping bits of human brain alive blurs the line of death and could necessitate new frameworks for consent or tissue ownership (32, 39). Although donors consent to research use of their tissue, they likely do not anticipate that their tissue would remain viable and functionally active ex vivo. Ensuring donors (or their families) are broadly informed about this possibility is one consideration. In addition, there may be an ethical duty of care for particularly long-lived cultures; for instance, if a slice culture unexpectedly remained viable for months or years, should there be guidelines on whether it is acceptable to maintain it indefinitely, or criteria for when it should be respectfully terminated? These questions currently have no definitive answers, but they underline the importance of ongoing ethical review as HBSC techniques evolve. In the absence of clear guidance for laboratories, the field, therefore, needs a globally recognized stewardship standard—ideally developed under the auspices of an international norm-setting body (e.g., UNESCO, WHO) or a professional consortium dedicated to human tissue research.

Such a framework would (i) recommend upper limits on culture duration, (ii) define “humane endpoints” based on future biomarkers of minimally viable network activity that could signal rudimentary perception or consciousness (e.g., persistent large-scale synchrony, complex cross-frequency coupling, or other validated signatures) (40, 41), and (iii) recommend ethically responsible disposal methods that promptly eliminate residual activity. Laboratories could then align local protocols with this consensus benchmark, and any breach of the agreed endpoints would trigger review by an independent ethics body, ensuring consistent respect for the unique moral status of living human neural tissue worldwide.

### Dual-use research of concern (DURC)

Another critical ethical dimension is the potential for DURC, defined as legitimate scientific research that could be misapplied to pose a significant threat to public health, safety, or security. This issue is particularly relevant in the context of high-content screening platforms that use human brain slice cultures. When combined with advanced gene-editing tools and AI-driven molecular design, such platforms could inadvertently accelerate the development of neuroactive or neurotoxic compounds with military or illicit potential (42). To mitigate these risks, experimental protocols involving pathogens, viral vectors, extensive synaptic reprogramming, or behavioral modulation should be subject to formal DURC assessment mechanisms. In the United States, guidance is provided by the National Science Advisory Board for Biosecurity (NSABB) through its Recommended Policy Guidance for Departmental Development of Review Mechanisms for Potential DURC (2007; updated 2016). Within the European Union, multiple complementary frameworks apply, including the EU Dual-Use Regulation (EU) 2021/821, as well as institutional ethics and biosafety review boards operating under GDPR and national biosecurity mandates (43).

As human neural systems research becomes increasingly translational and computationally enabled, alignment with these frameworks is essential, not only to safeguard ethical integrity but also to preempt international security concerns.

### Ethical advantage: animal reduction

Finally, it is worth noting an ethical benefit of HBSC research: its potential to reduce the use of animals in neuroscience research. By providing a human-specific model, HBSCs support the Replacement and Reduction principles of the 3Rs (replacement, reduction, and refinement of animal use). If certain experiments can be done on human slices instead of in vivo animal models, researchers can avoid subjecting animals to those interventions, which is a positive ethical outcome. This does not eliminate ethical concerns (because human tissue use has its own considerations, as discussed), but it shifts some of the burden away from animal testing.

In our view, the development of HBSCs is complementary to animal research, not a wholesale replacement, but as these human models improve, they may allow researchers to use fewer animals or skip certain animal experiments entirely when studying human-specific phenomena (Table 1).

**Table 1.** Key ethical considerations in HBSC research and recommended practices.

Ethical issue	Description/concern	Recommended practices
Informed consent	Explicit permission from donors or next of kin for using brain tissue in research, including clarity on tissue reuse, storage, data linkage, and withdrawal rights.	Use broad or dynamic consent models that detail experimental reuse, long-term viability, data integration (e.g., omics, imaging), and international sharing. Ensure ongoing donor communication when feasible.
Donor privacy and confidentiality	Protecting the personal identity and clinical/genetic data of tissue donors.	De-identify samples (coded identifiers); store data securely; comply with GDPR (EU), HIPAA (US), and institutional privacy frameworks. Include consent language on potential data linkage.
No impact on clinical care	Preventing interference of research goals with patient treatment or surgery.	Separate clinical decisions from research needs: only use tissue that would be removed as part of standard care; have clear hospital protocols to prevent any changes to surgery purely for research purposes.
Long-term stewardship	Maintaining viable brain slices raises moral questions about longevity, responsibility, and disposal.	Advocate for UNESCO/WHO-backed global guidelines. Define maximum culture durations and humane endpoints. Mandate disposal once activity thresholds are reached. Ethics review required for exceptional cases.
Dual-use research of concern (DURC)	Risk of misuse from high-throughput screening of brain tissue in combination with AI, gene-editing, or neuroactive agents.	Screen for DURC using NSABB guidelines (US) or EU Regulation 2021/821. Require red-team exercises, dual-use training, and secure storage of sensitive datasets. Align with institutional biosafety and biosecurity boards.
Animal replacement and 3Rs contribution	HBSCs may reduce the need for certain animal experiments, supporting ethical alternatives.	Promote HBSC use for human-specific phenomena. Emphasize integration with the 3Rs principle (replacement, reduction, and refinement). Evaluate when HBSCs can ethically replace in vivo testing.

With these ethical safeguards in place, the next challenge lies in harnessing the full potential of HBSCs through interdisciplinary innovation and global collaboration.

## Future Perspectives

Human brain slice cultures (HBSCs) are entering a transformative phase, with emerging technologies and interdisciplinary collaborations poised to expand their scientific and clinical relevance. Key future directions include the integration of spatial “omics” and high-content imaging, personalized drug testing, neurotoxicity screening, public engagement, and the formation of global collaborative and ethical frameworks.

### Integrating spatial omics and computational tools

As neuroscience increasingly demands high-resolution, human-relevant data, HBSCs offer a unique opportunity to integrate molecular, spatial, and functional insights in one platform. A major future direction is the incorporation of cutting-edge “omics” and imaging technologies to extract maximal information from each slice culture. Recent progress in spatial transcriptomics (such as spatial RNA-seq) and high-throughput proteomics/metabolomics can be leveraged to profile gene and protein expression directly within the 3D architecture of brain slices (13). Applying these spatial omics methods to HBSCs will allow researchers to map how different cell types (neurons, various glia, endothelial cells) in the slice respond to diseases or treatments at a molecular level, while preserving their spatial relationships. This could, for example, identify localized signaling changes in a microregion or track the spread of a neurodegenerative protein across a slice. In addition, high-content imaging combined with single-cell analysis is expected to become routine in slice research. By coupling organotypic cultures with automated microscopy and image analysis, one could monitor hundreds of individual cells over time to see how they change with drug exposure or disease progression. These approaches will generate massive datasets, and integrating them will require advanced computational tools. However, the payoff will be a holistic view of tissue biology: one can simultaneously observe functional outcomes (electrophysiological activity, cell survival) and the underlying molecular alterations, all in a human tissue context. This systems-level understanding could greatly facilitate the discovery of novel therapeutic targets and biomarkers that are relevant to human brain disorders (something much harder to achieve in animal or 2D models that lack the full extent of model complexity).

### Advancing personalized medicine and safety testing

A central application of HBSCs moving forward will be their integration into personalized medicine and safety pharmacology pipelines. These cultures offer a rare opportunity to test new therapies directly on healthy and diseased human brain tissue before clinical trials. For example, small biopsies of healthy cortex (e.g., from epilepsy surgeries) could be used to assess neurotoxicity profiles of novel compounds, allowing early elimination of candidates with human-specific off-target effects. Conversely, efficacy screening can be performed on patient-derived pathologic tissue (e.g., glioma or epileptic focus slices), guiding drug optimization and stratification. These “patient avatars” could be subjected to diverse treatment conditions in parallel. High-throughput drug screening on patient-specific slices, supported by automation and high-content analysis, enables rapid identification of effective therapies. Building on these methods, future HBSC platforms could serve as mini-clinical trials in a dish—evaluating safety and efficacy in human brain tissue with unprecedented resolution and translational value. Importantly, the future of this application hinges on protocol standardization, quality control metrics, and reproducibility frameworks.

### Public and patient engagement

As HBSC research moves forward, public engagement and patient involvement will become increasingly important. These cultures exist at the intersection of cutting-edge science and human biology, which can spark public curiosity and concern. Future initiatives should prioritize clear communication about how HBSCs are derived, their research applications, and how donor rights are preserved. This could include educational outreach, accessible publications, and codesigned consent forms. Furthermore, models of sustained donor engagement could be implemented, for example, anonymized updates on general research outcomes to donors or their families, or involvement of patient advisory boards in governance. Such engagement not only fosters trust but also strengthens the ethical foundations of HBSC research by treating donors as ongoing partners in discovery.

### Multidisciplinary and global collaboration

To fully realize the potential of HBSCs, coordinated efforts across disciplines and geographies are essential. Neuroscientists, neurosurgeons, neurologists, and neuropathologists must collaborate on optimized tissue access and characterization. Bioengineers can develop microfluidic perfusion platforms or biomimetic scaffolds to support long-term viability. Data scientists are increasingly crucial for analyzing and integrating multimodal datasets (e.g., electrophysiology, imaging, multi-omics). Computational neuroscientists can use HBSC data to validate in silico models of human brain function. International biobanks and culture consortia could democratize access to high-quality slices, enabling research in locations without direct surgical access. These networks could facilitate not only protocol sharing and training, but also the physical exchange of prepared cultures under ethical agreements. In parallel, a globally harmonized ethical framework will be required to address emerging challenges such as consciousness potential, consent longevity, and equitable data sharing. This framework should build upon discussions from earlier sections and be shaped by international agencies like UNESCO or WHO.

Together, these multidisciplinary and global collaborations will create a sustainable and ethically robust ecosystem for HBSC research, accelerating progress toward solving some of the most challenging problems in neuroscience and personalized medicine.

## Closing Remarks

HBSCs have advanced from a niche experimental setup to a versatile and powerful model at the frontier of neuroscience research. This field is still in its relative infancy, and many possibilities are yet to be fully explored. There remain uncertainties about the ultimate limits of the model—for example, how closely slice cultures can mirror the full complexity of an intact human brain, or how long we can maintain slices in a healthy, functional state. Nevertheless, recent and anticipated progress in bioengineering, molecular profiling, and interdisciplinary methods are poised to enhance the faithfulness and utility of HBSCs. Equally important, the ethical and regulatory frameworks surrounding this research are evolving to ensure responsible use of human tissue and to address novel questions proactively. As improvements continue to unfold, we expect HBSCs to play an increasingly critical role in advancing our understanding of brain function and disease. They stand to improve the translational pipeline by providing a more human-relevant test platform, potentially reducing the reliance on animal models and leading to more effective therapies for neurological and psychiatric disorders. In bridging the gap between in vitro and in vivo, and between laboratory research and patient care, human brain slice cultures represent a powerful tool to advance translational neuroscience while upholding ethical responsibility in human tissue research. We are optimistic that with collaborative effort and careful oversight, the next decade of HBSC research will yield breakthroughs that benefit both science and humanity.

## Supplementary Material

Reviewer comments
