# Supplementary material for: Human brain slice cultures: translational applications and ethical considerations
Source: Life Sci Alliance. 2025 Oct 9;8(12):e202403160. doi: 10.26508/lsa.202403160 (PMC12511760; doi:10.26508/lsa.202403160)
Supplement: Reviewer comments [file LSA-2024-03160_review_history.pdf]

Life Science Alliance

Human Brain Slice Cultures: Translational Applications and Ethical Considerations

Kevin Joseph, Ioannis Vasilikos, Juergen Grauvogel, Mukesch Johannes Shah, Peter Reinacher, Julia M Nakagawa, Ute Häussler, Jakob Straehle, Nicolas N Neidert, Panagiotis Fistouris, Matthias Schneider, Steven Sloan, Andreas Vlachos, Marco Prinz, Tobias Weiss, Volker A Coenen, Oliver Schnell, Ulrich G Hofmann, Jürgen Beck, Philipp Kellmeyer and Vidhya Madapusi Ravi
DOI: https://doi.org/10.26508/lsa.202403160

Corresponding author(s): Dr. Vidhya Madapusi Ravi (University Medical Center Freiburg)

Review Timeline:

Submission Date:	2024-12-05
Editorial Decision:	2025-02-06
Appeal Requested:	2025-05-11
Appeal Approved:	2025-05-21
Revision Received:	2025-05-22
Editorial Decision:	2025-07-15
Revision Received:	2025-08-01
Editorial Decision:	2025-09-04
Revision Received:	2025-09-15
Accepted:	

Scientific Editor: Tim Fessenden

Transaction Report:

(Note: With the exception of the correction of typographical or spelling errors that could be a source of ambiguity, letters and reports are not edited. The original formatting of letters and referee reports may not be reflected in this compilation.)

No full Peer Review Process File is available with this article, as the authors have chosen not to make the review process public in this case.

February 6, 2025

Re: Life Science Alliance manuscript #LSA-2024-03160

Dr. Vidhya Madapusi Ravi
University Medical Center Freiburg
Neurosurgery
Breisacher Str. 64
79106
Freiburg,, Baden Wurtemburg Freiburg
Germany

Dear Dr. Ravi,

Thank you for submitting your manuscript entitled "Human Brain Slice Cultures: Scientific Potential and Ethical Considerations" to Life Science Alliance. The manuscript has now been seen by expert reviewers, whose reports are appended below. Unfortunately, after an assessment of the reviewer feedback, our editorial decision is against publication in Life Science Alliance.

Given these Reviewer concerns, we are afraid that we are unable to proceed further with the paper. We are thus returning your manuscript to you at this time.

We are sorry our decision is not more positive, but hope that you find the reviews constructive. Of course, this decision does not imply any lack of interest in your work and we look forward to future submissions from your lab.

Thank you for your interest in Life Science Alliance.

Sincerely,

Eric Sawey, PhD
Executive Editor
Life Science Alliance
http://www.lsajournal.org

May 21, 2025

MS: LSA-2024-03160

Dr. Vidhya Madapusi Ravi
University Medical Center Freiburg
Neurosurgery
Breisacher Str. 64
79106
Freiburg,, Baden Wurtemburg Freiburg
Germany

Dear Dr. Ravi,

Your manuscript entitled "Human Brain Slice Cultures: Scientific Potential and Ethical Considerations" has now been reconsidered, and I am pleased to let you know that we have decided to reconsider our earlier decision and return your manuscript to the original reviewers.

Please use the following link to submit your revised manuscript:
https://lsa.msubmit.net/cgi-bin/main.plex?el=A3Na7BXc1A3CiUG5I5B9ftdNUrgudXPL76Oq7SGEnBl4gZ

Yours sincerely,

Tim Fessenden
Scientific Editor
Life Science Alliance

July 15, 2025

Re: Life Science Alliance manuscript #LSA-2024-03160R-A

Dr. Tobias Weiss
University Hospital of Zurich
q
Zurich Switzerland
Switzerland

Dear Dr. Weiss,

Thank you for submitting your revised manuscript entitled "Human Brain Slice Cultures: Scientific Potential and Ethical Considerations" to Life Science Alliance. We appreciate your patience during the unusually long re-review period. Only Reviewer 2 was available to evaluate this revised manuscript. While they continue to be overall positive about the work in terms of its suitability for Life Science Alliance, some important issues remain.

Our general policy is that papers are considered through only one revision cycle; however, given that the suggested changes are relatively minor, we are open to one additional short round of revision. Please note that I will expect to make a final decision without additional reviewer input upon re-submission.

Please submit the final revision within one month, along with a letter that includes a point by point response to the remaining reviewer comments.

To upload the revised version of your manuscript, please log in to your account: https://lsa.msubmit.net/cgi-bin/main.plex
You will be guided to complete the submission of your revised manuscript and to fill in all necessary information.

Please pay attention to adhere to our editorial requirements for revisions:

A. THESE ITEMS ARE REQUIRED FOR REVISIONS

-- A letter addressing the reviewers' comments point by point.

-- An editable version of the final text (.DOC or .DOCX) is needed for copyediting (no PDFs).

-- High-resolution figure, supplementary figure and video files uploaded as individual files: See our detailed guidelines for preparing your production-ready images, https://www.life-science-alliance.org/authors

-- Summary blurb (enter in submission system): A short text summarizing in a single sentence the study (max. 200 characters including spaces). This text is used in conjunction with the titles of papers, hence should be informative and complementary to the title and running title. It should describe the context and significance of the findings for a general readership; it should be written in the present tense and refer to the work in the third person. Author names should not be mentioned.

B. MANUSCRIPT ORGANIZATION AND FORMATTING:

Full guidelines are available on our Instructions for Authors page, https://www.life-science-alliance.org/authors

Thank you for this interesting contribution to Life Science Alliance.

Sincerely,

Tim Fessenden
Scientific Editor
Life Science Alliance

August 22, 2025

Re: Life Science Alliance manuscript #LSA-2024-03160RR

Dr. Vidhya Madapusi Ravi
University Medical Center Freiburg
Neurosurgery
Breisacher Str. 64
79106
Freiburg,, Baden Wurtemburg Freiburg
Germany

Dear Dr. Ravi,

Thank you for submitting your revised manuscript entitled "Human-to-Human Brain Slice Cultures: Translational Applications and Ethical Considerations" to Life Science Alliance. While we are overall positive about the work in terms of its suitability for Life Science Alliance, some important issues remain.

Please submit the final revision within one month, along with a letter that includes a point by point response to the remaining reviewer comments.

To upload the revised version of your manuscript, please log in to your account: https://lsa.msubmit.net/cgi-bin/main.plex
You will be guided to complete the submission of your revised manuscript and to fill in all necessary information.

Please pay attention to adhere to our editorial requirements for revisions:

A. THESE ITEMS ARE REQUIRED FOR REVISIONS

-- A letter addressing the reviewers' comments point by point.

-- An editable version of the final text (.DOC or .DOCX) is needed for copyediting (no PDFs).

-- High-resolution figure, supplementary figure and video files uploaded as individual files: See our detailed guidelines for preparing your production-ready images, https://www.life-science-alliance.org/authors

-- Summary blurb (enter in submission system): A short text summarizing in a single sentence the study (max. 200 characters including spaces). This text is used in conjunction with the titles of papers, hence should be informative and complementary to the title and running title. It should describe the context and significance of the findings for a general readership; it should be written in the present tense and refer to the work in the third person. Author names should not be mentioned.

B. MANUSCRIPT ORGANIZATION AND FORMATTING:

Full guidelines are available on our Instructions for Authors page, https://www.life-science-alliance.org/authors

Thank you for this interesting contribution to Life Science Alliance.

Sincerely,

Tim Fessenden
Scientific Editor
Life Science Alliance

September 15, 2025

RE: Life Science Alliance Manuscript #LSA-2024-03160RRR

Dr. Vidhya Madapusi Ravi
University Medical Center Freiburg
Neurosurgery
Breisacher Str. 64
79106
Freiburg,, Baden Wurtemburg Freiburg
Germany

Dear Dr. Ravi,

Thank you for submitting your Review entitled "Human Brain Slice Cultures: Translational Applications and Ethical Considerations". We appreciate your patience while we evaluated this newly revised manuscript, which we agree has resolved all remaining issues. It is a pleasure to let you know that your manuscript is now accepted for publication in Life Science Alliance. Congratulations on this interesting work.

The final published version of your manuscript will be deposited by us to PubMed Central upon publication.

Your manuscript will now progress through copyediting and proofing. It is journal policy that authors provide original data upon request.

Reviews, decision letters, and point-by-point responses associated with peer-review at Life Science Alliance will be published, alongside the manuscript. If you do want to opt out of having the reviewer reports and your point-by-point responses displayed, please let us know immediately.

IMPORTANT: If you will be unreachable at any time, please provide us with the email address of an alternate author. Failure to respond to routine queries may lead to unavoidable delays in publication.

Scheduling details will be available from our production department. You will receive proofs shortly before the publication date. Only essential corrections can be made at the proof stage so if there are any minor final changes you wish to make to the manuscript, please let the journal office know now.

DISTRIBUTION OF MATERIALS:
Authors are required to distribute freely any materials used in experiments published in Life Science Alliance. Authors are encouraged to deposit materials used in their studies to the appropriate repositories for distribution to researchers.

You can contact the journal office with any questions, contact@life-science-alliance.org

Again, congratulations on a very nice paper. I hope you found the review process to be constructive and are pleased with how the manuscript was handled editorially. We look forward to future exciting submissions from your lab.

Sincerely,

Tim Fessenden
Scientific Editor
Life Science Alliance
